# Engagement in Transformational Leadership by Teachers Influences the Levels of Self-Esteem, Motor Self-Efficacy, Enjoyment, and Intention to Be Active in Physical Education Students

**DOI:** 10.3390/sports12070191

**Published:** 2024-07-15

**Authors:** Carolina Sánchez-García, Rafael E. Reigal, Jacobo Hernández-Martos, Antonio Hernández-Mendo, Veronica Morales-Sánchez

**Affiliations:** 1 Department of Social Psychology, Social Anthropology, Social Work and Social Services, University of Málaga, 29010 Málaga, Spain; casanchez@uma.es (C.S.-G.); rafareigal@uma.es (R.E.R.); mendo@uma.es (A.H.-M.); 2 Faculty of Psychology, University of Málaga, 29071 Málaga, Spain; jacobohm@uma.es

**Keywords:** wellness, fitness, education, positive habits, leadership, confidence, identity

## Abstract

In the school context, the teacher–student interaction style plays a pivotal role in ensuring optimal adolescent functioning. Over recent years, the significance of transformational teacher leadership in fostering student engagement and positive development has been underscored. This study aimed to examine the correlations between transformational leadership and adolescent self-esteem, motor self-efficacy, enjoyment in physical education classes, and intention for future physical activity. This research used an associative and predictive strategy. A total of 429 adolescents from Málaga, aged between 14 and 16 years (M = 14.97; SD = 0.85), participated in this research, which employed an associative, comparative, and predictive approach. The Transformational Teaching Questionnaire (TTQ), Motor Self-Efficacy Scale (MSES), Intrinsic Satisfaction in Sport Instrument (SSI-EF), and Intention of Being Physically Active Scale (MIFA) were utilized for variable assessment. Correlation, Multiple Regression, and cluster analyses revealed statistically significant correlations between transformational leadership and self-esteem, motor self-efficacy, enjoyment, and future activity intention. Specifically, it was revealed that motivational inspiration predicts self-esteem, motor self-efficacy, enjoyment, and intention for physical activity, whereas intellectual stimulation predicts motor self-efficacy, enjoyment, and intention for physical activity. Notably, the transformational leadership factors of intellectual stimulation and motivational inspiration were observed to predict scores across other variables, particularly enjoyment in physical education classes, both overall and by gender. These findings suggest that transformational leadership in physical education classes can significantly enhance student experiences, thereby promoting adherence to physical activity and healthy lifestyles among adolescents.

## 1. Introduction

Physical activity is essential for well-being and health promotion [1]. Its benefits are manifold, including stress and anxiety reduction, as well as the promotion of emotional well-being [2]. Moreover, it facilitates social interaction and cooperation among individuals, strengthening self-esteem and motor self-efficacy [3]. Therefore, the promotion of regular physical activity and the development of healthy lifestyles should be a primary objective for our society. However, during adolescence, it has been observed that the recommended levels of physical activity are not always met, reducing the potential for physical activity to act as a health-promoting element [4]. Due to the high prevalence of sedentary behavior in adolescence, schools can be excellent promoters of active lifestyles. Indeed, physical education classes are considered an ideal space to provide positive experiences of physical practice [4]. On one hand, in these classes, it is ensured that adolescents will engage in physical activity regularly as they are required to attend them. On the other hand, if students enjoy these classes, they could be encouraged to engage in physical activity during leisure time [5].

To ensure the effectiveness of these classes in promoting physical activity, it is necessary for students to have positive experiences, with their perception of their teachers’ behavior style being essential [5]. The interaction between teacher and student in the realm of physical education is proposed as a crucial factor in creating an active learning environment where students feel supported to explore their motor skills, fostering commitment to physical exercise and promoting healthy lifestyles among adolescents [6]. Thus, the style of leadership exercised by the teacher is considered particularly determinant, given its implications for student behavior [7]. Leadership style refers to the way teachers act, which is based on their understanding of their teaching role and their expectations for students, while also involving the values and ethical principles of each professional [8]. Various leadership styles have been studied in the field of education, as proposed by Britwum (2021) [9], including authoritarian, democratic, and laissez-faire leadership. However, two new leadership styles that could be relevant in school contexts have recently been highlighted: transactional leadership and transformational leadership [10].

Several recent studies on leadership in students have highlighted the importance of the transformational leadership style, in contrast to other styles, for student development and their commitment to academic tasks [10,11]. It has been observed that the engagement of teachers in transformational leadership, characterized by charisma, motivational inspiration, individualized consideration, and intellectual stimulation, significantly impacts students’ self-esteem and motor self-efficacy [12,13]. Additionally, this type of leadership fosters a motivational environment that promotes enjoyment during classes, thereby increasing the likelihood of adherence to physical activity practice both within and outside the school environment [14]. Thus, understanding how to apply transformational leadership during physical education classes could be a key component in creating environments where students actively engage in physical activity [15].

As Rangel (2023) [16] outlines, the transformational leadership model presents numerous advantages, as the leader becomes a role model, fostering cooperative collaboration wherein socio-personal skills such as adolescents’ self-esteem are developed. Self-esteem can be defined as the evaluation we make of our own worth, based on the set of thoughts, emotions, sensations, and experiences accumulated throughout life [17]. Within the framework of transformational leadership in physical education classes, it is observed that the development of self-esteem through the transformational interaction style plays a crucial role as a precursor to adolescents’ inclination towards future physical activity [18]. This positive sentiment associated with physical practice will contribute to the development of a favorable attitude towards it, impacting both the activity performed in the school context and in other physical practice contexts [19].

Among other aspects, the transformational leadership style of physical education teaching fosters adolescents’ perception of motor self-efficacy [20], which is the confidence in one’s own physical abilities [21]. This is essential for developing a satisfactory experience and increasing the likelihood of adhering to exercise practice [22]. Since students perceive motivation inspiration and individual consideration during physical education classes, their perception of motor self-efficacy could be enhanced. Thus, if students perceive themselves as motorically effective in overcoming physical exercises, there is a possibility that behaviors related to future active lifestyles may increase [23]. 

Transformational leadership used by physical education teachers stimulates enjoyment during classes, a key factor highlighted in previous research [24]. Enjoyment in physical education classes is defined as the sensation of enjoyment while engaging in physical activity [5], promoted through individual consideration and motivational inspiration from the teacher [25]. Specifically, a transformational teacher fosters a positive and enjoyable atmosphere by demonstrating confidence in students’ abilities to overcome physical challenges, refraining from passing judgments, and instead cultivating motivation and security. When students experience positive sensations of enjoyment in physical education classes, they are more likely to continue practicing physical activity in the future [26]. Thus, promoting an atmosphere of enjoyment in physical education classes through transformational leadership is crucial for fostering active lifestyles in adolescents.

Similarly, authors like Castillo et al. (2020) [18] have studied the relationships between transformational leadership and active lifestyles modulated by motivational climates during physical education classes. Specifically, they have demonstrated that transformational leadership contributes to adherence to active lifestyles. Along this line, Moreno Casado et al. (2022) [27] stated that the engagement of teachers in transformational leadership influences students’ enjoyment. However, there is a lack of recent research that has highlighted a connection between transformational leadership, motor self-efficacy, self-esteem, and enjoyment in physical education classes to promote the maintenance of active lifestyles in adolescents. Recent previous research in this area is scarce, as most studies relate some of these variables individually; there are no previous studies that relate the same variables as the present study where the sample is composed of adolescents. Nevertheless, the results of this research will provide statistically relevant findings that will contribute to the comprehensive development of this academic field. Therefore, the aim of this research was to establish the relationships between transformational leadership, self-esteem, motor self-efficacy, enjoyment, and intention to continue practicing physical activity in adolescents. Therefore, our main aim was to determine the existence of significant relationships between transformational leadership in physical education teachers and students’ self-esteem, motor self-efficacy, enjoyment, and intention to be active.

## 2. Materials and Methods

### 2.1. Research Design

This research used a cross-sectional design, particularly, an associative and predictive strategy, according to Ato et al. (2013) [28]. This approach begins with gathering data on relevant variables, such as transformational leadership exhibited by physical education teachers, and student outcomes like self-esteem, motor self-efficacy, enjoyment, and intention to be physically active. Through correlation analyses, it assesses whether there are significant correlations between these variables. Additionally, regression analyses are employed to predict how changes in one variable (e.g., transformational leadership) might affect outcomes in others (e.g., student motivation and activity levels).

### 2.2. Participants

This study included 429 participants, with 47.8% (n = 205) being male and 52.2% (n = 224) female. The ages of the sample ranged from 14 to 16 years (M = 14.97; SD = 0.845). To be included in this research, students were required to attend physical education classes regularly, be competent in reading and comprehending questions related to the study, not have recent injuries that prevented their participation in physical education, and have been enrolled in school for a certain period. Eligible participants were individuals who regularly attended physical education classes, were enrolled in the second, third, or fourth year of Compulsory Secondary Education, and were aged between 14 and 16 years.

### 2.3. Instruments

The Transformational Teaching Questionnaire (TTQ) [29] was employed to assess teachers’ transformational leadership, utilizing the Spanish version developed by Álvarez et al. (2018) [30]. This questionnaire gauges students’ perceptions of their physical education teachers’ behaviors associated with the transformational leadership style. It comprises 16 items grouped into four factors: individualized consideration (e.g., ‘Shows that s/he cares about me’), idealized influence (e.g., ‘Acts as a person that I look up to’), intellectual stimulation (e.g., ‘Creates lessons that really encourage me to think’), and inspirational motivation (e.g., ‘Is enthusiastic about what I am capable of achieving’). Responses are recorded on a Likert-type scale, ranging from one (never) to five (always). The internal consistency for this research, measured by Cronbach’s alpha (1951) [31], demonstrated values of 0.88 for individualized consideration, 0.85 for idealized influence, 0.83 for intellectual stimulation, and 0.90 for inspirational motivation.

The Rosenberg Self-Esteem Scale (RSE) [32,33] explores an individual’s overall self-attitude, encompassing both positive and negative self-esteem. With 10 items, respondents rate their perspectives on a Likert-type scale from 1 (strongly disagree) to 4 (strongly agree), which is structured around two factors: positive (e.g., “I feel as valuable as others”) and negative self-esteem (e.g., “I wish I had more respect for myself”). Negative items are represented by items 2, 5, 8, 9, 10 and positive items are represented by items 1, 3, 4, 6, 7. For this study, we used only the factor positive self-esteem. The internal consistency analysis showed a value of 0.85 for positive self-esteem and 0.79 for negative self-esteem.

The Motor Self-Efficacy Scale (MSES) [34], an adaptation of the Baessler and Schwarzer (1996) [35] General Self-Efficacy Scale, assesses the perception of competence in coping with motor tasks. With 10 items (e.g., “During a sports game, I can solve a problem even if someone opposes me.”), respondents use a Likert-type scale from 1 (strongly disagree) to 4 (strongly agree). The internal consistency analysis yielded a value of 0.87.

The Intrinsic Satisfaction in Sport Instrument (SSI-EF) [36] measures enjoyment in physical education (PE). The Spanish version evaluates intrinsic satisfaction through eight items, focusing on enjoyment and boredom factors [4,37]. Enjoyment is reflected in five statements such as “I typically find pleasure in my PE classes”. On the other hand, boredom is characterized by three expressions like “During PE, I often find myself eagerly anticipating the end of the class”. Respondents use a Likert-type scale from one (do not agree at all) to four (strongly agree). For this study, only the enjoyment factor was considered, with a Cronbach’s alpha value of 0.88

The Intention of Being Physically Active Scale (MIFA) [38], an adapted version of the scale from Hein et al. (2004) [39], evaluates individuals’ intent for future physical activity beyond the school setting. Comprising 10 items (e.g., “I am interested in improving my physical fitness”), respondents use a Likert-type scale from 1 (strongly disagree) to 5 (strongly agree). The internal consistency analysis showed a Cronbach’s alpha value of 0.81. 

### 2.4. Procedure

The sample selection was carried out across various educational institutions in the city of Málaga, Spain. These schools shared notable similarities in terms of socioeconomic characteristics, curriculum plans, resources, and time allocated to physical education. Authorization was requested from the management of each institution, and once obtained, informed consent was obtained from parents/guardians for their children’s participation in the study. The data were collected by the researchers who are listed as authors of the present article. In the informed consent form given to the parents, the objective of the article was detailed, as well as the questionnaires that were going to be administered. This consent form had to be signed by the parents and by the participants of the study themselves. Subsequently, a detailed explanation of the study’s purpose was provided to the students, emphasizing its entirely voluntary nature and the guarantee of data confidentiality. After expressing their willingness to participate, additional information was provided to the students. Data collection took place during a physical education session in the classroom, with students completing the questionnaires in approximately one hour. A researcher provided detailed instructions on the process of completing of the tests to minimize potential errors. Throughout the questionnaire administration, the team was available to address any queries that may arise.

Throughout the research process, adherence to the principles enshrined in the Declaration of Helsinki was ensured. Approval from the ethics committee of the University of Málaga was obtained to conduct this research (CEUMA: 209-2023-H).

### 2.5. Data Analysis

The data underwent both descriptive and inferential analyses. Mean values, standard deviations, skewness, and kurtosis were estimated. Additionally, the normality of the data was assessed using the Kolmogorov–Smirnov test. Subsequently, correlation analyses were conducted using the Pearson correlation coefficient (±0.01 to ±0.19 = very weak correlation; ±0.20 to ±0.39 = weak correlation; ±0.40 to ±0.59 = moderate correlation; ±0.60 to ±0.79 = high correlation) [40]. Furthermore, Multiple Regression Analysis (using intro regression) was performed to analyze the predictive capacity of transformational leadership on self-esteem, motor self-efficacy, enjoyment, and intention to be active. Finally, cluster analyses (k-means) were employed to generate clusters based on dimensions of transformational leadership, aiming to extract groups with homogeneous characteristics and observe differences in self-esteem, motor self-efficacy, enjoyment, and intention to be active. Moreover, effect size was calculated using Cohen’s d statistic (≈0.20: small, ≈0.50: medium, and ≈0.80: large) [41,42]. ANOVA was used for mean contrasts, and pairwise comparisons were conducted using the Bonferroni statistic. The data were processed using the SPSS software package version 25.

## 3. Results

Table 1 displays the mean values, standard deviation, skewness, and kurtosis for the study variables. Additionally, the Kolmogorov–Smirnov test was conducted, yielding appropriate values in all cases (*p* > 0.05). Therefore, the distributions of the variables did not present normality issues. 

Table 1 presents various statistical measures, including mean, standard deviation, skewness, kurtosis, and the Kolmogorov–Smirnov test, for the overall sample, as well as for boys and girls separately. It was established that the variables follow a normal distribution. In Table 2, correlations (Pearson) between the analyzed variables are shown. The analyses highlighted statistically significant relationships in all variables, both in the total sample and by gender. It was evidenced that self-esteem is positively related to enjoyment, the four components of transformational leadership, self-efficacy, and intention to continue practicing, both in the overall sample and in boys and girls. Also, it was observed that the strongest correlations were found between the transformational leadership and enjoyment variables, indicating a moderately high correlation, according to Evans (1996) [40].

Table 3, Table 4 and Table 5 display the Multiple Regression Analysis using the intro method, where collinearity analysis was conducted using the Variance Inflation Factor (VIF) and Tolerance tests, obtaining values between 0.23 and 7.45 for Tolerance, and between 1.00 and 4.30 for VIF. Thus, according to several authors, these results are considered acceptable in the context of regression analysis [43,44]. This suggests that there is no appreciable multicollinearity issue in the data, supporting the reliability of the results obtained in the regression analysis. Variables that do not appear in the different cases were excluded due to their lack of relevance (*p* > 0.05). Each of the generated models meets the required conditions to be considered acceptable, including linearity in the relationship between predictor variables and the criterion, as well as homoscedasticity and a normal distribution of residuals. It is observed that the mean value of the residuals is close to 0, with a standard deviation of practically 1 (0.99). Additionally, the values obtained for Durbin–Watson are adequate, ranging between 1.56 and 2.34 [45].

Models were developed to explain each of the variables analyzed in the study (self-esteem, motor self-efficacy, enjoyment, and intention to be active) in the total sample. These models encompass variation ranging from 11% to 41%. According to the results, motivational inspiration was a predictor of self-esteem (R = 0.34; adjusted R^2^ = 0.11; F = 14.24; *p* < 0.001), motor self-efficacy (R = 0.43; adjusted R^2^ = 0.18; F = 24.51; *p* < 0.01), enjoyment (R = 0.65; adjusted R^2^ = 0.41; F = 75.40; *p* < 0.001), and intention to be active (R = 0.36; adjusted R^2^ = 0.12; F = 15.30; *p* < 0.05). Additionally, intellectual stimulation was a predictor of motor self-efficacy (R = 0.43; adjusted R^2^ = 0.18; F = 24.51; *p* < 0.01), enjoyment (R = 0.65; adjusted R^2^ = 0.41; F = 75.40; *p* < 0.001), and intention to be active (R = 0.36; adjusted R^2^ = 0.12; F = 15.50; *p* < 0.05). Finally, in the total sample, individual consideration was a predictor of enjoyment (R = 0.65; adjusted R^2^ = 0.41; F = 75.40; *p* < 0.001).

For boys, a set of explanatory models was established for each variable examined in this study: self-esteem, motor self-efficacy, enjoyment, and intention to be active. These models explain the variation in a range of from 15% to 48%. The results indicate that in boys, intellectual stimulation was a predictor of self-esteem (R = 0.44; adjusted R^2^ = 0.18; F = 11.93; *p* < 0.01), motor self-efficacy (R = 0.50; adjusted R^2^ = 0.24; F = 16.63; *p* < 0.001), and enjoyment (R = 0.71; adjusted R^2^ = 0.49; F = 49.94; *p* < 0.01), and motivational inspiration was a predictor of self-esteem (R = 0.19; adjusted R^2^ = 0.18; F = 11.93; *p* < 0.05), enjoyment (R = 0.44; adjusted R^2^ = 0.18; F = 49.94; *p* < 0.01), and intention to be active (R = 0.40; adjusted R^2^ = 0.15; F = 9.72; *p* < 0.01).

In the case of girls, explanatory models were formulated for each variable analyzed in this research: self-esteem, motor self-efficacy, enjoyment, and intention to be active. These models provide an explanation of the variation that ranges from 8% to 36%. Considering the results, it was evident that motivational inspiration was a predictor of self-esteem (R = 0.33; adjusted R^2^ = 0.09; F = 6.70; *p* < 0.001), motor self-efficacy (R = 0.41; adjusted R^2^ = 0.15; F = 11.05; *p* < 0.001), and enjoyment (R = 0.62; adjusted R^2^ = 0.37; F = 33.43; *p* < 0.01). Additionally, intellectual stimulation was a predictor of intention to be active (R = 0.33; adjusted R^2^ = 0.10; F = 6.85; *p* < 0.05).

### Cluster Analysis

Through cluster analysis (K-means), three groups were generated based on the dimensions of transformational leadership. Each case was well classified as the maximum distance of each one from the center of its group, which was always less than the distances between the centers of each cluster. The three groups (cluster 1, n = 144; cluster 2, n = 222; cluster 3 = 63) were characterized by (G1) moderate scores in the dimensions of transformational leadership, (G2) high scores in the dimensions of transformational leadership, and (G3) low scores in the dimensions of transformational leadership.

In Table 6, the means and standard deviations of the three groups are shown. Additionally, the results of skewness (−2.14, 0.16) and kurtosis (−0.90, 1.72) in the three groups, as well as the Kolmogorov–Smirnov test (*p* > 0.05) showed that the distributions did not present normality problems. Likewise, an analysis of variance (ANOVA) was conducted to check for differences between groups. As observed, there were statistically significant differences between groups in all variables, favoring the group with higher transformational leadership values. Post hoc comparisons were made using the Games–Howell statistic since the test of variance homogeneity (Levene) was significant in all cases (*p* < 0.05).

## 4. Discussion

The purpose of this study was to establish the relationships between transformational leadership, self-esteem, motor self-efficacy, enjoyment, and intention to continue practicing physical activity in adolescents during physical education classes. The results obtained in this study are supported by previous scientific literature [24]. It is widely known that the leadership style of physical education teachers, especially the transformational interaction style, is one of the main mechanisms of influence on physical practice in adolescents [6].

Firstly, through Pearson correlations, it was evidenced that self-esteem was positively related to enjoyment, the four components of transformational leadership, self-efficacy, and intention to continue practicing, both in the overall sample and in boys and girls [16,18,26]. Therefore, when the teacher promotes individual consideration, idealized influence, intellectual stimulation, and motivational inspiration, students will feel higher self-esteem, more motor efficacy, and the intention to be active, and perceive the class as fun. Hence, students with higher self-esteem will enjoy physical education classes more, and feel more motor self-efficacious, and consequently, will want to continue practicing physical activity [18]. This relationship will be positively modulated by transformational leadership. If a teacher employs a transformational leadership style, their students will develop better self-esteem, have a high perception of self-efficacy, and enjoy physical education classes more; thus, they will have higher intentions to continue practicing physical activity in the future [18,19,20,23]. Conversely, without transformational leadership, students may have low self-esteem and motor self-efficacy, reducing the intention to practice physical activity and limiting adherence to physical education classes.

Secondly, the results obtained from the Multiple Regression models reaffirm the findings obtained from the correlations, but they specify which factor of transformational leadership predicts each of the variables in the present study. It was found that motivational inspiration served as a predictor of self-esteem, motor self-efficacy, enjoyment, and intention to be active [18]. Likewise, intellectual stimulation acted as a predictor of motor self-efficacy, enjoyment, and intention to be active. Lastly, in the overall sample, individual consideration was identified as a predictor of enjoyment. Regarding individual consideration, it is proposed as a predictor of enjoyment in the overall sample. These findings could be explained by the fact that, in the context of physical education classes, the teacher’s ability to inspire critical thinking among adolescents could positively affect self-esteem, motor self-efficacy, and enjoyment, contributing to adherence to physical activity [24,26]. Additionally, personalized treatment could contribute to creating a more positive and stimulating environment for adolescents, which, in turn, would contribute to their overall enjoyment of physical activities.

In the gender-based analysis of Multiple Regression, it was observed that there are two factors of transformational leadership that have a greater influence than others, specifically, intellectual stimulation and motivational inspiration. In the case of intellectual stimulation, it was a predictor of self-esteem, motor self-efficacy, and enjoyment in boys, and of enjoyment and intention to be active in girls. It is possible that the intellectual stimulation provided by the teacher has a more direct impact on the self-confidence and enjoyment of physical activities in boys, while in girls, it may be more linked to the intention to actively participate. Regarding the motivational inspiration variable, it was a predictor of enjoyment and intention to be active in boys, and of self-esteem, motor self-efficacy, and enjoyment in girls. This suggests that in boys, the motivational inspiration provided by the teacher can have an impact on the perception of physical activity as attractive and on the desire to actively engage in classes. Meanwhile, in girls, feeling inspired, motivated, and supported by their teacher may lead to an increase in self-esteem and confidence in their motor skills [46]. It is important to highlight that both variables (motivational inspiration and intellectual stimulation) predict enjoyment, which suggests that regardless of gender, adolescents feel more enjoyment when they perceive classes as intellectually stimulating and when they provide motivational inspiration.

Thirdly, along the same line, the results obtained through the K-means cluster analysis reveal effective segmentation into three distinct groups based on the dimensions of transformational leadership. It is observed that the differences between the group with high scores in transformational leadership (G2) and the other two groups (G1 and G3) are consistently significant in all variables considered, indicating that transformational leadership is positively associated with enjoyment, motor self-efficacy, self-esteem, and intention to be active. Additionally, it was found that the differences between the groups with moderate (G1) and low (G3) scores in transformational leadership were statistically significant in most variables, suggesting a gradual correlation between the level of transformational leadership and self-esteem, motor self-efficacy, intention to be active, and enjoyment in adolescents [24].

Therefore, the findings of this article are in line with previous literature suggesting that a higher level of transformational leadership is related to better self-esteem, motor self-efficacy, and intention to be active, but above all, to enjoyment [18,47]. When interpreting these results, it is important to consider some limitations of this research. Firstly, the lack of an experimental design incorporating specific control and experimental groups according to leadership styles could have affected the precision of the conclusions. Likewise, the omission of considering external variables, such as socioeconomic factors, along with the absence of a prolonged follow-up, constitute limitations that require attention. Additionally, the restriction of the sample to adolescents in third and fourth grade of secondary education raises questions about generalization to the population as a whole, indicating the need to diversify the age of the sample in future research.

Despite the limitations presented, our results indicate that transformational interaction styles in physical education classes could play a crucial role in determining engagement in physical activity, thus promoting healthy lifestyles among adolescents. Furthermore, they suggest that the adoption of transformational interaction styles during physical education classes stimulates enjoyment, raises self-esteem, and strengthens perceived self-efficacy in adolescents during adolescence. Therefore, at a societal level, it would be important to create training programs in transformational leadership for physical education teachers with the aim of promoting changes in students’ lifestyles, thus fostering adherence to physical activities.

## 5. Conclusions

This study highlights the importance of transformational leadership in the context of physical education classes to promote healthy lifestyles among adolescents. The results suggest that the implementation of transformational interaction styles by physical education teachers can stimulate enjoyment, elevate self-esteem, and strengthen perceived self-efficacy in adolescents, which, in turn, can increase their engagement in physical activity. These findings have important implications for the design of interventions aimed at promoting transformational leadership styles in contexts that foster physical activity and the well-being of adolescents, with the goal of achieving adherence to exercise and active lifestyles.

## Figures and Tables

**Table 1:** Descriptive statistics of total, male, and female samples.

	Total Sample n = 429				Male n = 205				Female n = 224			
	M	SD	S	K	M	SD	S	K	M	SD	S	K
1.Individual consideration	4.05	0.96	−0.97	0.25	4.09	0.91	−0.93	0.31	4.02	1.00	−0.97	0.15
2. Idealized influence	3.76	0.98	−0.77	−0.02	3.83	0.91	−0.76	−0.06	3.68	1.03	−0.72	−0.19
3. Intellectual stimulation	3.71	0.93	−0.63	0.11	3.75	0.89	−0.43	0.35	3.67	0.96	−0.75	0.34
4. Motivational inspiration	3.99	1.00	−0.89	0.07	4.00	0.96	−0.86	−0.57	3.98	1.02	−0.91	0.08
5. Enjoyment	3.39	0.66	−1.19	0.89	3.51	0.58	−1.35	1.50	3.27	0.70	−1.03	0.41
6. Motor self-efficacy	3.11	0.53	−0.50	0.55	3.24	0.48	−0.29	−0.38	2.99	0.54	−0.57	0.86
7. Self-esteem	3.23	0.68	−1.14	0.94	3.47	0.57	−1.26	1.54	3.10	0.73	−0.96	0.36
8. Intention to be active	3.90	0.86	−0.74	0.04	4.08	0.80	−0.81	−0.162	3.72	0.88	−0.67	0.99

Notes: M = Mean; SD = Standard Deviation; S = Skewness; K = Kurtosis; K-S = Kolmogorov–Smirnov.

**Table 2:** Correlations of total, male, and female samples.

	Total Sample				Male				Female			
	E	MSE	SE	ITBA	E	MSE	SE	ITBA	E	MSE	SE	ITBA
1. Individual consideration	0.60 ***	0.34 ***	0.26 ***	0.28 ***	0.64 **	0.39 ***	0.35 ***	0.33 ***	0.57 ***	0.31 ***	0.21 ***	0.24 ***
2. Idealized influence	0.59 ***	0.36 ***	0.28 ***	0.29 ***	0.65 ***	0.39 ***	0.36 ***	0.31 ***	0.54 ***	0.32 ***	0.21 ***	0.25 **
3. Intellectual stimulation	0.59 ***	0.41 ***	0.28 ***	0.33 ***	0.65 ***	0.48 ***	0.41 ***	0.34 ***	0.54 ***	0.35 ***	0.18 ***	0.31 ***
4. Motivational inspiration	0.62 ***	0.40 ***	0.34 ***	0.34 ***	0.67 ***	0.44 ***	0.40 ***	0.39 ***	0.59 ***	0.39 ***	0.30 ***	0.29 ***

Notes: E: enjoyment; MSE: motor self-efficacy; SE: self-esteem; ITBA: intention to be active. ** *p* < 0.01; *** *p* < 0.001.

**Table 3:** Multiple Regression Analysis (intro) in total sample.

Sample	Criteria	R	R^2^ Adj.	D-W	Predictors	Beta	T	Tolerance	VIF
Total	Self-esteem	0.34	0.11	1.97	Individual consideration	−0.10	−0.97	0.19	5.15
					Idealized influence	−0.03	−0.30	0.18	5.60
					Intellectual stimulation	0.06	0.77	0.31	3.21
					Motivational inspiration	0.40 ***	3.96	0.20	5.03
	Motor self-efficacy	0.43	0.18	1.85	Individual consideration	−0.09	−0.90	0.19	5.15
					Idealized influence	−0.07	−0.70	0.18	5.60
					Intellectual stimulation	0.28 ***	3.53	0.31	3.21
					Motivational inspiration	0.33 **	3.33	0.20	5.03
	Enjoyment	0.65	0.41	2.06	Individual consideration	0.16	1.86	0.19	5.15
					Idealized influence	0.02	0.20	0.18	5.60
					Intellectual stimulation	0.21 **	3.20	0.31	3.21
					Motivational inspiration	0.30 ***	3.63	0.20	5.03
	Intention to be active	0.36	0.12	1.93	Individual consideration	−0.07	−0.69	0.19	5.15
					Idealized influence	−0.08	−0.71	0.18	5.60
					Intellectual stimulation	0.21 *	2.56	0.31	3.21
					Motivational inspiration	0.30 *	2.93	0.120	5.03

Note: D-W = Durbin–Watson; VIF = Variance Inflation Factor; * *p* < 0.05; ** *p* < 0.01; *** *p* < 0.001.

**Table 4:** Multiple Regression Analysis (intro) in male sample.

Sample	Criteria	R	R^2^ Adj.	D-W	Predictors	Beta	T	Tolerance	VIF
Male	Self-esteem	0.44	0.18	1.94	Individual consideration	−0.07	−0.47	0.19	5.19
					Idealized influence	−0.05	−0.30	0.18	5.60
					Intellectual stimulation	0.28 **	2.44	0.31	3.27
					Motivational inspiration	0.29 *	2.03	0.20	4.91
	Motor self-efficacy	0.50	0.24	1.86	Individual consideration	−0.03	−0.19	0.19	5.19
					Idealized influence	−0.16	−1.10	0.18	5.60
					Intellectual stimulation	0.42 ***	3.78	0.31	3.27
					Motivational inspiration	0.27	2.02	0.20	4.91
	Enjoyment	0.71	0.49	1.88	Individual consideration	0.10	0.88	0.19	5.19
					Idealized influence	0.10	0.83	0.18	5.60
					Intellectual stimulation	0.26 **	2.83	0.31	3.27
					Motivational inspiration	0.30 **	2.74	0.20	4.91
	Intention to be active	0.40	0.15	1.97	Individual consideration	−0.01	−0.07	0.19	5.19
					Idealized influence	−0.14	−0.90	0.18	5.60
					Intellectual stimulation	0.14	1.20	0.31	3.27
					Motivational inspiration	0.41 **	2.86	0.20	4.91

Note: D-W = Durbin–Watson; VIF = Variance Inflation Factor; * *p* < 0.05; ** *p* < 0.01; *** *p* < 0.001.

**Table 5:** Multiple Regression Analysis (intro) in female sample.

Sample	Criteria	R	R^2^ Adj.	D-W	Predictors	Beta	T	Tolerance	VIF
Female	Self-esteem	0.33	0.09	2.08	Individual consideration	−0.09	−0.65	0.19	5.14
					Idealized influence	−0.13	−0.88	0.17	5.74
					Intellectual stimulation	−0.09	−0.78	0.31	3.18
					Motivational inspiration	0.57 ***	3.90	0.19	5.24
	Motor self-efficacy	0.41	0.15	1.89	Individual consideration	−0.11	−0.80	0.19	5.14
					Idealized influence	−0.13	−0.90	0.17	5.74
					Intellectual stimulation	0.18	1.63	0.31	3.18
					Motivational inspiration	0.46 ***	3.29	0.19	5.24
	Enjoyment	0.62	0.37	2.04	Individual consideration	0.21	1.75	0.19	5.14
					Idealized influence	−0.11	−0.83	0.17	5.74
					Intellectual stimulation	0.17	1.83	0.31	3.18
					Motivational inspiration	0.37 **	3.07	0.19	5.24
	Intention to be active	0.33	0.10	2.07	Individual consideration	−0.12	−0.82	0.19	5.14
					Idealized influence	−0.10	−0.66	0.17	5.74
					Intellectual stimulation	0.26 *	2.32	0.31	3.18
					Motivational inspiration	0.28	1.92	0.19	5.24

Note: D-W = Durbin–Watson; VIF = Variance Inflation Factor; * *p* < 0.05; ** *p* < 0.01; *** *p* < 0.001.

**Table 6:** Cluster analysis (k-means).

	G1		G2		G3			G1 vs. G2	G1 vs. G3	G2 vs. G3
	M	SD	M	SD	M	SD	F			
Enjoyment	3.24	0.57	3.71	0.42	2.61	0.77	112.54 ***	−0.47 ***^c^	0.63 ***^c^	1.10 ***^a^
Motor self-efficacy	3.15	0.59	3.40	0.62	2.84	0.83	33.55 ***	−0.29 ***^a^	0.21 *^a^	0.51 ***^b^
Self-esteem	2.99	0.43	3.29	0.49	2.78	0.60	19.87 ***	−0.24 ***^b^	0.31 *^a^	0.56 ***^c^
Intention to be active	3.73	0.82	4.14	0.75	3.41	1.03	23.77 ***	−0.40 ***^b^	0.32 ^a^	0.73 ***^c^

Note: * *p* < 0.05; *** *p* < 0.001. D de Cohen, a ≈ 0.20: small, b ≈ 0.50: medium y c ≈ 0.80: big.

## Data Availability

Data are available upon request to the authors.
